# Optimizing disinfection and adventitious shoot propagation in *Cyclocarya paliurus* stem tissue culture: enzymatic and flavonoid metabolomics insights

**DOI:** 10.1186/s12870-026-08646-z

**Published:** 2026-04-01

**Authors:** Ying Feng, Yongshan Chen, Zhixiong Guo, Jinghua Xu

**Affiliations:** 1 School of Resources and Environmental Science, Quanzhou Normal University, Quanzhou, 362000 China; 2 College of Horticulture, Fujian Agriculture and Forestry University, Fuzhou, 350000 China

**Keywords:** *Cyclocarya paliurus*, Tissue culture, Adventitious shoot, Propagation

## Abstract

**Background:**

*Cyclocarya paliurus* (Batal.) Iljinskaja is a medicinal and endangered woody plant, but shoot culture is challenged by higher contamination and lower propagation efficiency.

**Results:**

This study evaluated the effects of various sterilization and propagation treatments on shoot culture, and further analyzed enzymatic activity and flavonoid metabolomics at 20, 40, 60, and 80 days of culture using UPLC-MS. The optimal disinfection method-cold storage at 4 °C for 8 days, followed by immersion in warm water at 37 °C, and sterilization with 0.1% mercuric chloride for 5 min-resulted in a contamination rate of 12.72%. The highest propagation coefficient (5.91-fold) was achieved on ½ MS medium supplemented with 0.5 mg/L 6-BA, 0.01 mg/L TDZ, 0.3 mg/L GA₃ and 0.02 mg/L IBA at 60 days. Enzymatic activity followed the order: SOD > POD > PPO > PAL. A total of 151 flavonoid-related metabolites were identified and classified into 11 categories, including flavonols (28.48%), flavones (18.54%), flavanones (10.60%), benzoic acid and its derivatives (6.62%), flavanols (5.96%), flavanonols (5.30%), isoflavanones (4.64%) and others. Metabolite content varied with culture duration, with catechin (a flavanol) showing the highest content (> 1000 µg/g) during shoot culture.

**Conclusion:**

Pretreatment of “lower-temperature storage and warm-water immersion” efficiently reduced stem contamination, and significant associations existed between enzymatic activity, flavonoid profiles, and the propagation of adventitious shoot. These findings offer a promising strategy to improve shoot propagation, in vitro conservation and utilization in *C.* species.

**Supplementary Information:**

The online version contains supplementary material available at 10.1186/s12870-026-08646-z.

## Background

*Cyclocarya paliurus* (Batal.) Iljinskaja (*C. paliurus*), a member of the genus *Cyclocarya* in the Juglandaceae family, is a rare, endangered species native to China [1], and has been traditionally consumed as “sweet tea” for over 1,000 years. In recent years, *C. paliurus* has gained recognition as a medicinal resource due to its health-promoting properties, including anti-aging, anti-inflammatory, anticancer, and anti-diabetic effects [2, 3]. Hence, it has received official approval as a novel food ingredient from the National Health and Family Planning Commission of China [4].

However, natural populations of *C. paliurus* have suffered from overexploitation and anthropogenic disturbances [5]. Additionally, its propagation is hindered by a low seed germination rate (0.1%-0.2%) and a high proportion of empty seeds. Vegetative propagation through cuttings is also difficult due to poor adventitious root formation [5, 6], rendering conventional propagation methods unsuitable for propagating this species. Plant tissue culture offers a promising alternative for rapid and large-scale propagation under controlled, sterile condition independent of seasonal constraints [7]. By implementing tissue culture for *C. paliurus*, the micropropagation of this species can protect this rare, endangered species and reduce strain on natural populations.

Shoot culture of *C. paliurus* from stems has been extensively explored [6, 8]. However, two major challenges have seriously hindered the effective propagation in *C.* species. The first is the extremely high contamination rate of explants (above 95%), especially when using field-grown stems as explants. This is primarily attributed to the presence of persistent endophytes and pathogens deep within the stem tissue [9], which remain unaffected by surface sterilization agents such as mercuric chloride (HgCl₂) or sodium hypochlorite (NaClO) [10]. Therefore, it is crucial to obtain an effective explant sterilization for a successful in vitro propagation protocol. Various sterilization strategies have been applied to various species to minimize or eradicate explant contamination. These strategies involve pretreating explants through various techniques like UVC irradiation pretreatment [11], cold treatment with antioxidant solutions [12], heat treatment ranging from 35 °C to 42 °C [7, 13], or multi-stage disinfection for stem explants [14]. Additionally, the control of explant contamination is proven to be affected by the source and type of explant, along with sterilization duration [7, 15, 16]. Despite this extensive research, the needle sterilization technique for field-grown *C. species* stems remains unexplored.

The second challenge involves shoot death and leaf abscission, which are prone to lower propagation efficiency of adventitious shoots [6, 8]. This is affected by external factors like plant growth regulators (PGRs) and internal factors such as enzymatic activity and metabolite compounds during culture. For external factors, a successful shoot propagation is heavily reliant on PGRs combination [16, 17], unsuitable PGRs combinations can hinder shoot development and propagation [15, 18]. CKs (like 6-BA, TDZ), GAs and auxin (like IBA) are commonly used in various tissue culture to prevent organ abscission and death [19]. For instance, a combination of 6-BA (0.5–1.5 mg/L) and IBA (1.0–2.0 mg/L) has proven effective in shoot multiplication of *C. paliurus* [8], GA_3_ (1.0–2.0 mg/L) aids in shoot development and inhibits tissue death [17]. In terms of internal factors, prolonged culture duration results in variations in physiological activities, such as the excessive accumulation and fluctuation of phenolic metabolites and antioxidant enzymatic activities (e.g., Superoxide dismutase (SOD), Polyphenol oxidase (PPO), Phenylalanine ammonialyase (PAL), Peroxidase (POD)) [19, 20], further intensifying shoot senescence and death. To date, no effective solutions have been established for overcoming these issues in shoot culture from field-grown stems of *C. paliurus*.

Numerous researches have investigated bioactive compounds in *C. paliurus*, with some flavonoids found in higher concentrations than other compounds (e.g. triterpenoids and polysaccharides) in leaf extracts [21, 22]. Flavonoids, which are derived from the phenylpropanoid pathway, encompass a diverse group including flavonols, flavones, flavanones, and isoflavones [23]. These compounds play vital roles in plant physiologies and possess antioxidant properties by scavenging free radicals [24], potentially mitigating browning and death of adventitious shoots. Chen et al. [25]. reported that flavonols were the predominant flavonoids, followed by flavones and isoflavones, mainly present as O-glycosides. However, limited knowledge is obtained on flavonoid dynamics during shoot culture, particularly their correlation with shoot propagation.

In vitro culture is essential for the propagation of this rare and endangered species, which holds considerable economic and medical value. Therefore, this study aims to: (1) evaluate contamination rates of field-grown stems under different treatment conditions (cold storage duration, water type comparison, and sterilization duration); (2) assess effects of various propagation treatments (PGRs combination and culture duration) on adventitious shoot growth; (3) investigate flavonoid-related metabolomics and enzymatic activity across different culture durations. These approaches are anticipated to significantly improve the propagation coefficient of *C. paliurus*, thereby supporting its wider cultivation and conservation of its diversity.

## Materials and methods

### Explant collection and culture initiation

**Fig. 1:**
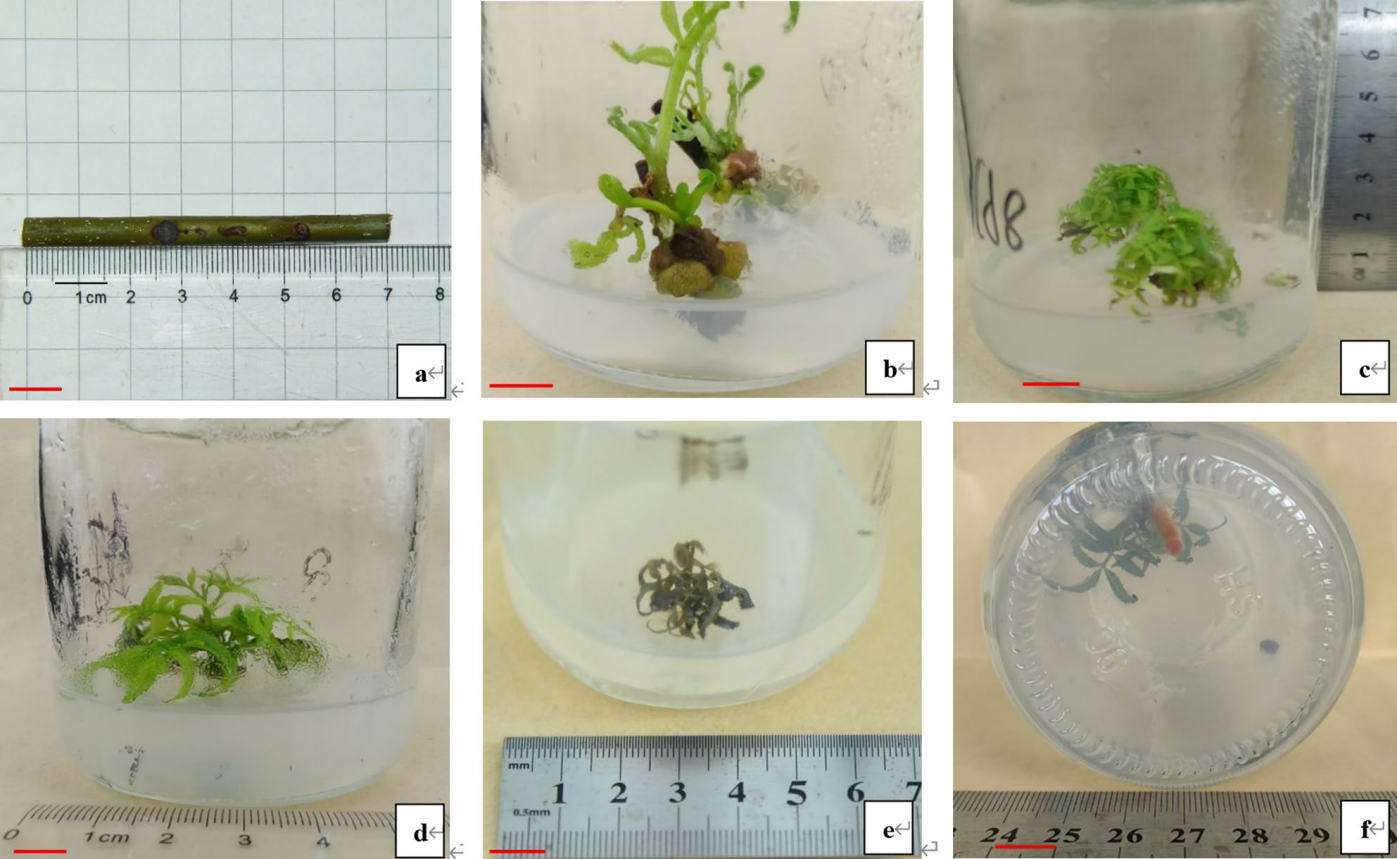
Shoot culture of *Cyclocarya paliurus*. **a** denotes mature stem from *Cyclocarya paliurus* plant; **b** denotes shoot development; **c** and **d** denote adventitious shoot propagation; **e** denotes adventitious shoot death; **f** denotes root growth. Bars = 1.0 cm

Mature stems were from over 20-year-old *Cyclocarya paliurus* (Batal.) Iljinskaja trees, and provided by Fengtian National Forestry Farm in Quanzhou City, Fujian Province (E117°40′37″, N25°15′53″. No wild material was collected; therefore, no permits or voucher specimens were required.). Fully lignified stems were cut into node segments (approximately 7–8 cm in length). Each segment contained 4–5 adventitious shoots, and was used as explant (Fig. 1a).

Unless otherwise specified, all culture media contained 30 g/L sucrose and 6 g/L agar, adjusted to pH 6.0, and autoclaved at 121 °C for 20 min. The basic medium was ½ MS medium containing half-strength major elements, calcium salts, and iron salts, while maintaining the same concentration of trace elements and organic elements as MS medium. Culture condition was maintained under cool white fluorescent light (283.02 µmol/m²·s) with a 12 h light/12 h dark photoperiod at 22 ± 2 °C. All experiments were conducted using a single factor randomized design. Each treatment included 30 samples, placed 2 shoots per bottle, with three replicates.

### Explant disinfection treatment

Explants underwent various disinfection protocols, and then rinsed with sterilized water. After 20 days of culture, the optimal disinfection method was determined based on the contamination rate (the number of contaminated explants / total number of explants).

The treatments were as follows: (1) Cold storage duration: Explants were stored at 4 °C for 0 (as control), 2, 4, 6, 8, or 10 days. After storage, they were rinsed in purified-water (from a reverse osmosis system) at 37 °C for 5 min, repeated 3–4 times. Explants were then disinfected with 75% ethanol for 30 s, followed by treatment with 0.1% mercuric chloride (HgCl₂) for 5 min. (2) Water type comparison: Explants stored at 4 °C for 8 days were rinsed with different water types — purified-water at 37 °C, sterilized water, and purified-water— each for 5 min, repeated 3–4 times. They were then disinfected with 75% ethanol for 30 s and treated with 0.1% HgCl₂ for 5 min. (3) Sterilization duration: Explants stored at 4 °C for 8 days were rinsed in purified-water at 37 °C for 5 min (3–4 repetitions), then disinfected with 75% ethanol for 30 s and treated with 0.1% HgCl₂ for varying durations (1, 3, 5, 7, 9, or 11 min).

### Culture for adventitious shoot propagation

#### PGRs treatments for shoot development

Explants without contamination were transferred to culture media to induce shoot development. Two media groups were used: (1) TDZ treatment: ½ MS medium supplemented with TDZ (0.1, 0.3, or 0.5 mg/L) and 0.01 mg/L NAA. (2) TDZ + GA₃ treatment: ½ MS medium supplemented with TDZ (0.1, 0.3, or 0.5 mg/L), 0.01 mg/L NAA, and 0.1 mg/L GA₃. After 30 days of culture, the optimal medium for shoot development was selected based on shoot development rate.

#### PRGs treatments for adventitious shoot propagation

Shoots were excised from nodes and transferred to ½ MS medium containing different PGRs combinations: (1) 6-BA variation: 6-BA (0.3, 0.5, or 0.7 mg/L) with 0.01 mg/L TDZ, 0.3 mg/L GA₃, and 0.02 mg/L IBA. (2) TDZ variation: TDZ (0, 0.01, or 0.03 mg/L) with 0.5 mg/L 6-BA, 0.3 mg/L GA₃, and 0.02 mg/L IBA. (3) GA₃ variation: GA₃ (0, 0.1, 0.3, or 0.5 mg/L) with 0.5 mg/L 6-BA, 0.01 mg/L TDZ, and 0.02 mg/L IBA. (4) IBA variation: IBA (0, 0.02, or 0.04 mg/L) with 0.5 mg/L 6-BA, 0.01 mg/L TDZ, and 0.3 mg/L GA₃. A comprehensive overview of PGRs combinations in each treatment was found in Tables 1, 2, 3 and 4. After 30 days of culture, the appropriate PGRs combination for adventitious shoot propagation was determined according to propagation coefficient and average height of adventitious shoots.

**Table 1:** Effects of 6-BA treatments on adventitious shoot growth

	6-BA (mg/L)	TDZ (mg/L)	GA_3_ (mg/L)	IBA (mg/L)	Average shoot height (cm)	Propagation coefficient (fold)
1	0.3	0.01	0.3	0.02	0.16 ± 0.01^b^	4.04 ± 0.28^ab^
2	0.5	0.01	0.3	0.02	1.86 ± 0.19^a^	4.48 ± 0.45^a^
3	0.7	0.01	0.3	0.02	0.29 ± 0.04^b^	3.16 ± 0.38^b^

Different superscripted letters denote significant difference (α = 0.05) between each other with Tukey’s HSD test for means (*p* ≤ 0.05). The same superscripted letter denote no difference between each other. All basic medium is ½ MS medium

**Table 2:** Effects of TDZ treatments on adventitious shoot growth

	6-BA (mg/L)	TDZ (mg/L)	GA_3_ (mg/L)	IBA (mg/L)	Average shoot height (cm)	Propagation coefficient (fold)
1	0.5	0	0.3	0.02	0.32 ± 0.01^b^	2.48 ± 0.54^b^
2	0.5	0.01	0.3	0.02	1.86 ± 0.19^a^	4.48 ± 0.45^a^
3	0.5	0.03	0.3	0.02	0.25 ± 0.05^b^	2.59 ± 0.11^b^

Different superscripted letters denote significant difference (α = 0.05) between each other with Tukey’s HSD test for means (*p* ≤ 0.05). The same superscripted letters denote no difference between each other. All basic medium is ½ MS medium

**Table 3:** Effects of GA₃ treatments on adventitious shoot growth

	6-BA (mg/L)	TDZ (mg/L)	GA_3_ (mg/L)	IBA (mg/L)	Average shoot height (cm)	Propagation coefficient (fold)
1	0.5	0.01	0	0.02	0.37 ± 0.03^bc^	2.51 ± 1.31^b^
2	0.5	0.01	0.1	0.02	0.32 ± 0.03^c^	2.90 ± 0.16^ab^
3	0.5	0.01	0.3	0.02	1.86 ± 0.19^a^	4.48 ± 0.45^a^
4	0.5	0.01	0.5	0.02	0.58 ± 0.02^b^	2.97 ± 0.04^ab^

Different superscripted letters denote significant difference (α = 0.05) between each other with Tukey’s HSD test for means (*p* ≤ 0.05). The same superscripted letter denotes no difference between each other. All basic medium is ½ MS medium

**Table 4:** Effects of IBA treatments on adventitious shoot growth

	6-BA (mg/L)	TDZ (mg/L)	GA_3_ (mg/L)	IBA (mg/L)	Average shoot height (cm)	Propagation coefficient (fold)
1	0.5	0.01	0.3	0	0.48 ± 0.06^c^	3.42 ± 0.56^a^
2	0.5	0.01	0.3	0.02	1.85 ± 0.19^a^	4.48 ± 0.45^a^
3	0.5	0.01	0.3	0.04	1.12 ± 0.07^b^	3.62 ± 0.43^a^

Different superscipted letters denote significant difference (α = 0.05) between each other with Tukey’s HSD test for means (*p* ≤ 0.05). The same superscripted letter denotes no difference between each other. All basic medium is ½ MS medium

#### Effects of culture duration on adventitious shoot propagation

Adventitious shoots were cultured on ½ MS medium supplemented with 0.5 mg/L 6-BA, 0.01 mg/L TDZ, 0.3 mg/L GA₃, and 0.02 mg/L IBA, with varying durations (20, 30 (as control), 40, 60, and 80 days) to assess the influence of culture duration on adventitious shoot propagation. And the suitable culture duration was determined by propagation coefficient, death rate and average height of adventitious shoots.

### Root induction and plantlet transplantation

Following the method of Wu et al. [6], shoots approximately 1.0 cm in height were transferred to ½ MS medium supplemented with 0.5 mg/L NAA and 0.5 mg/L IBA for root induction. After 30 days of culture, rooted plantlets were transplanted into a substrate consisting of peat soil and perlite at a ratio of 10:1.

### Enzyme activity determination

Samples were obtained from adventitious shoots cultured on ½ MS medium supplemented with 0.5 mg/L 6-BA, 0.01 mg/L TDZ, 0.3 mg/L GA₃, and 0.02 mg/L IBA at different culture durations (20, 30 (as control), 40, 60, and 80 days), then immediately frozen with liquid nitrogen, and finally stored at -80℃ for subsequent enzymatic activity analysis.

Enzymes (SOD, PAL, PPO, POD) were extracted from frozen-samples following Feng et al. [26] and Li [27]. Briefly, samples were grounded and mixed with 15 mL of 0.05 mol/L phosphate buffer (PBS, pH 7.8), kept at 4 °C for 15 min, and centrifuged at 4 °C for 10 min at 11,000 *g*. The supernatant (enzymatic extract, E) was used to determine enzymatic activity assays using the method of Li [27], which included the following enzymes: (1) SOD activity: a reaction mixture containing enzymatic extract (E1) from E, PBS, methionine, EDTA, riboflavin, and nitro-blue tetrazolium. Samples were exposed to 4,000 lx light for 20 min, and absorbance was measured at 560 nm. (2) POD activity: measured using guaiacol and H₂O₂ in PBS. Enzymatic extract (E2) from E was added, and absorbance was recorded at 470 nm over 3 min. (3) PPO activity: assessed using catechol in PBS. Enzymatic extract (E3) from E was incubated at 37 °C for 10 min, then trichloroacetic acid was added, and the mixture was centrifuged. Absorbance was measured at 420 nm. (4) PAL activity: extracted with PBS (pH 8.8) and PVP for obtaining enzymatic extract (E4). Enzymatic extract (E5) from E4 was incubated with phenylalanine and PBS at 30 °C, and absorbance was measured at 290 nm. Enzymatic activities were calculated based on the following formula [26, 27].

$$\begin{aligned} \begin{array}{l}\mathrm{S}\mathrm{O}\mathrm{D}\:\mathrm{a}\mathrm{c}\mathrm{t}\mathrm{i}\mathrm{v}\mathrm{i}\mathrm{t}\mathrm{y}\:(\mathrm{U}/\mathrm{g}\cdot \mathrm{F}\mathrm{W})=\frac{{(\mathrm{A}}_{\mathrm{c}\mathrm{k}}-{\mathrm{A}}_{\mathrm{E}})\times\:{\mathrm{V}}_{\mathrm{E}}}{0.5\times\:{\mathrm{A}}_{\mathrm{c}\mathrm{k}}\times\:\mathrm{W}\times\:{\mathrm{V}}_{\mathrm{E}1}}\\\:\mathrm{P}\mathrm{O}\mathrm{D}\:\mathrm{a}\mathrm{c}\mathrm{t}\mathrm{i}\mathrm{v}\mathrm{i}\mathrm{t}\mathrm{y}\:(\mathrm{U}/\mathrm{g}\cdot \mathrm{m}\mathrm{i}\mathrm{n})=\frac{{\triangle\mathrm{D}}_{470}\times\:{\mathrm{V}}_{\mathrm{E}}}{0.01\times\:\mathrm{t}\times\:\mathrm{W}\times\:{\mathrm{V}}_{\mathrm{E}2}}\\\:\mathrm{P}\mathrm{P}\mathrm{O}\:\mathrm{a}\mathrm{c}\mathrm{t}\mathrm{i}\mathrm{v}\mathrm{i}\mathrm{t}\mathrm{y}\:(\mathrm{U}/\mathrm{g}\cdot \mathrm{m}\mathrm{i}\mathrm{n})=\frac{{\triangle\mathrm{D}}_{420}\times\:{\mathrm{V}}_{\mathrm{E}}}{0.01\times\:\mathrm{t}\times\:\mathrm{W}\times\:{\mathrm{V}}_{\mathrm{E}3}}\\\:\mathrm{P}\mathrm{A}\mathrm{L}\:\mathrm{a}\mathrm{c}\mathrm{t}\mathrm{i}\mathrm{v}\mathrm{i}\mathrm{t}\mathrm{y}\:(\mathrm{U}/\mathrm{g}\cdot \mathrm{m}\mathrm{i}\mathrm{n})=\frac{{\triangle\mathrm{D}}_{290}\times\:{\mathrm{V}}_{\mathrm{E}4}}{0.01\times\:\mathrm{t}\times\:\mathrm{W}\times\:{\mathrm{V}}_{\mathrm{E}5}}\end{array} \end{aligned}$$

A_ck_ and A_E_ are the absorbance of the control and samples at 560 nm, respectively; △D_470_ is the change of absorbance measured at 470 nm; △D_420_ is the change of absorbance measured at 420 nm; △D_290_ is the change of absorbance measured at 290 nm; V_E_ is total volume of E, mL; V_E1_ is total volume of E1, mL; V_E2_ is total volume of E2, mL; V_E3_ is total volume of E3, mL; V_E4_ is total volume of E4, mL; V_E5_ is total volume of E5, mL; t is reaction time, min; W is leaf fresh weight (g).

### Flavonoid metabolomics evaluation

#### Flavonoid extraction

Samples were obtained from adventitious shoots cultured on ½ MS medium supplemented with 0.5 mg/L 6-BA, 0.01 mg/L TDZ, 0.3 mg/L GA₃, and 0.02 mg/L IBA at different culture durations (20, 40, 60, and 80 days), and then frozen to dry. Frozen-dried samples were extracted with 0.5 mL of 70% methanol containing 0.4 mg/L internal standard ([2H6]Daidzein, IsoReag, Purity ≥ 97%), and then sonicated for 30 min, finally centrifuged at 12,000 *g* for 5 min at 4 °C. The supernatant was filtered through a 0.22 μm membrane for further analysis.

#### Flavonoid determination

Flavonoid-related metabolites were analyzed using ultra-performance liquid chromatography (UPLC, ExionLC™ AD) coupled with tandem mass spectrometry (MS, QTRAP^®^ 6500+). Chromatographic separation was performed on a Waters Acquity UPLC HSS T3 C18 column (1.8 μm, 100 mm × 2.1 mm) at 40 °C. The mobile phase consisted of: water with 0.05% formic acid (A); acetonitrile with 0.05% formic acid (B); flow rate: 0.35 mL/min; and gradient: 0–1 min: 90% A: 10% B; 2–9 min: 80% A: 20% B; 10–12.5 min: 30% A: 70% B;12.6 – 13.5 min: 5% A: 95% B; 13.6–16 min: 90% A: 10% B. Injection volume: 2 µL. MS parameters: Source temperature: 550 °C, Ion spray voltage: 5500 V (positive) / 4500 V (negative), and curtain gas: 35 psi.

Quality control (QC) samples were run every 10 samples to monitor instrument stability. Data were collected in multiple reaction monitoring (MRM) mode, identified using Analyst 1.6.3, and quantified with Multiquant 3.0.3. Data were log-transformed and mean-centered for orthogonal partial least squares-discriminant analysis (OPLS-DA) using the MetaboAnalyst R package.

#### Analysis of differentially accumulated flavonoid metabolites

Differentially accumulated metabolites (DAMs) were identified based on variable importance in projection (VIP > 1) and absolute log₂ fold change (|log₂FC| ≥ 1.0), with VIP values extracted from OPLS-DA. DAMs were annotated using the KEGG Compound Database and mapped to KEGG pathways. Enrichment analysis was performed using Metabolite Set Enrichment Analysis (MSEA).

### Statistical analysis

Results are presented as mean ± standard deviation (SD). One-way analysis of variance (ANOVA) was performed to assess differences among treatments at α = 0.05. Tukey’s Highly Significant Differences (HSD) test (*p* ≤ 0.05) was used for post hoc comparisons in SPSS software (version 19.0). Correlation analysis between flavonoid-related metabolites, enzymatic activities, and growth indices was conducted using Origin software (version 9.1, Northampton, MA01060, USA). Co-occurrence network analysis was further performed using Gephi software (version 0.9.2, WebAtlas, France) to visualize correlation between flavonoid metabolites, enzymatic activities and growth parameters.

## Results and analysis

### Effect of cold storage duration on explant contamination

**Fig. 2:**
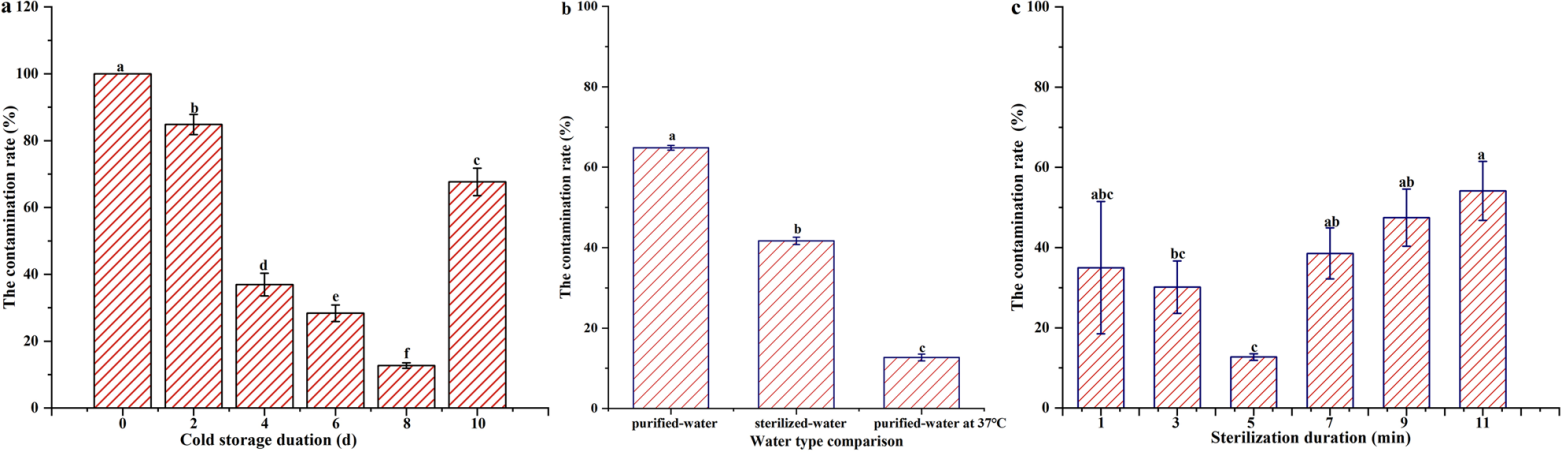
Effect of different disinfection treatments on mature stems in Cyclocarya paliurus. **a** denotes different cold storage duration treatments; **b** denotes water type comparison; **c** denotes different sterilization duration treatments. Different letters denote significant difference (α=0.05) between each other with Tukey’s HSD test for means

Explants stored for 0 days (control) exhibited a 100% contamination rate. Compared to the control, storage at 4 °C significantly reduced contamination, contamination rates decreased by 15.15% (2 days), 63.06% (4 days), 71.64% (6 days), 87.29% (8 days), and 32.34% (10 days) (Fig. 2a). The lowest contamination rate was at 8 days, with a contamination rate of only 12.72%. Compared to an 8-day storage duration, contamination rates increased by 85.02% (2 days), 65.59% (4 days), 55.17% (6 days), and 81.21% (10 days).

### Effect of water treatment on explant contamination

Contamination rates varied significantly among water treatments, following the order: purified-water > sterilized water > purified-water at 37 °C (Fig. 2b). Compared to purified-water treatment, contamination rates decreased by 35.66% (sterilized water), and 80.39% (purified-water at 37 °C).

### Effect of sterilization duration on explant contamination

Contamination rates varied with sterilization duration, showing an initial decrease from 1 to 5 min and then a significant increase from 7 to 11 min. The 5-minute treatment reached the lowest rate, reducing contamination rate by 63.66% compared to 1-minute treatment. Nevertheless, no difference in contamination rate was observed beyond 5 min of sterilization duration (Fig. 2c).

### Effect of PGRs treatments on shoot development

After 10 days of culture, top shoots began to develop, with leaf expansion and stem elongation. Typically, 2–4 shoots developed simultaneously, with the top shoot growing faster than the others (Fig. 1b).

The suitable medium for shoot development was the ½ MS medium supplemented with 0.3 mg/L TDZ, 0.1 mg/L GA₃ and 0.01 mg/L NAA, reaching the highest development rate (51.04%). However, shoot development rate under TDZ + GA₃ treatments increased in comparison to TDZ treatments. The increase was 30.37% for 0.1 mg/L TDZ + GA₃, 18.92% for 0.3 mg/L TDZ + GA₃ and 42.41% for 0.5 mg/L TDZ + GA₃, compared to the same TDZ concentration under TDZ treatments.

Shoot development rate varied with TDZ concentrations. For TDZ treatments, shoot development rate initially increased and then decreased as TDZ concentration increased, but no difference was observed among three TDZ concentrations. For TDZ + GA₃ treatments, shoot development rate consistently increase with higher TDZ concentrations, but there was no difference among them (Fig. S1).

### Effects of PGRs treatments on adventitious shoot propagation

The optimal medium for adventitious shoot propagation was ½ MS supplemented with 0.5 mg/L 6-BA, 0.01 mg/L TDZ, 0.3 mg/L GA₃, and 0.02 mg/L IBA, yielding the highest propagation coefficient and shoot height (Table 1). However, adventitious shoots cultured on media containing varying concentrations of 6-BA, TDZ, GA₃, and IBA exhibited different growth patterns. Some shoots propagated without stem elongation, while others showed both propagation and stem elongation (Fig. 1c and d).

### Effects of 6-BA and TDZ on adventitious shoot propagation

Shoot height and propagation coefficient varied with 6-BA and TDZ concentrations (Table 1). For 6-BA treatments, shoot height was significantly inhibited at 0.3 and 0.7 mg/L compared to 0.5 mg/L. Shoot propagation coefficient differed substantially between 0.5 and 0.7 mg/L, but showed no difference between 0.3 and 0.5 mg/L.

As to TDZ treatment, shoot height was the lowest (0.25 cm) at 0.03 mg/L, and increased by 28.0% (0.0 mg/L) and 644.0% (0.01 mg/L) compared to TDZ at the concentration of 0.03 mg/L. Shoot propagation coefficient increased by 80.65% (0.01 mg/L) and 4.44% (0.03 mg/L) compared to TDZ at the concentration of 0.0 mg/L (Table 2).

### Effects of GA₃ on adventitious shoot propagation

GA₃ significantly promoted adventitious shoot growth compared to medium without GA₃ (Table 3). Both shoot height and propagation coefficient increased with rising GA₃ concentrations. While shoot height showed significant difference among three GA₃ concentrations, shoot propagation coefficient remained relatively stable.

### Effects of IBA on adventitious shoot propagation

In the absence of IBA, shoot height was only 0.48 cm (Table 4). The addition of IBA significantly promoted shoot height, increasing by 287.5% (0.02 mg/L) and 133.33% (0.04 mg/L) compared to IBA at the concentration of 0.0 mg/L (Table 4). However, no significant difference was observed in shoot propagation coefficient across IBA concentrations.

### Effects of culture duration on adventitious shoot propagation

Shoot propagation coefficient increased progressively with longer culture durations, peaking at 60 days-1.32 times higher than at 30 days (Table 5). A similar result was observed in shoot height, but no difference existed between 60 days and 80 days. Adventitious shoot death began after 50 days of culture (Fig. 1e), with shoot death rate at 60 days was only 13% of that at 80 days.

**Table 5:** Effects of different culture durations on adventitious shoot growth and enzymatic activity

Culture duration (day)	Propagation coefficient (fold)	Shoot height (cm)	Shoot death (%)	SOD (U/g FW)	PAL (U/(g·min))	PPO (U/(g·min))	POD (U/(g·min))
20	2.13 ± 0.45^d^	1.37 ± 0.18^c^	0 ± 0^c^	460.72 ± 162.90^ab^	2.13 ± 0.42^b^	4.11 ± 0.84^b^	109.72 ± 5.55^ab^
30	4.48 ± 0.45^c^	1.86 ± 0.19^bc^	0 ± 0^c^	520.73 ± 3.71^ab^	5.56 ± 1.27^b^	15.2 ± 5.25^b^	98.00 ± 8.08^bc^
40	4.88 ± 0.20^bc^	2.12 ± 0.17^b^	0 ± 0^c^	303.83 ± 68.75^b^	5.17 ± 0.75^b^	10.00 ± 2.00^b^	40.40 ± 2.10^d^
60	5.91 ± 0.19^ab^	3.16 ± 0.32^a^	7.96 ± 1.00^b^	573.55 ± 60.45^a^	5.56 ± 1.27^b^	43.93 ± 4.81^a^	126.51 ± 12.15^a^
80	6.33 ± 0.52^a^	3.51 ± 0.41^a^	63.15 ± 1.44^a^	536.33 ± 71.16^ab^	9.64 ± 2.68^a^	34.13 ± 12.93^a^	85.03 ± 7.58^c^

Different superscripted letters denote significant difference (α = 0.05) between each other with Tukey’s HSD test for means (*p* ≤ 0.05). The same superscripted letter denotes no difference between each other. Adventitious shoots were cultured on ½ MS medium supplemented with 0.5 mg/L 6-BA, 0.01 mg/L TDZ, 0.3 mg/L GA₃, and 0.02 mg/L IBA

### Root induction and plantlet transplantation

During root induction, stem bases expanded and red roots formed (Fig. 1f). Rooting rate reached 28.25%, with only 1–2 roots per plantlet. However, no plantlets survived transplantation into a peat-perlite substrate (10:1), and leaf abscission was observed during transplantation.

### Enzymatic activity at different culture durations

Activities of SOD, PAL, PPO, and POD varied across culture durations, with the order: SOD > POD > PPO > PAL (Table 5). At 60 days, SOD, PPO, and POD activities increased by 10.14%, 188.99%, and 29.09%, respectively, compared to 30 days. PAL activity showed no significant change between at 30 days and 60 days.

### Flavonoid metabolite variations at different culture durations

UPLC-MS analysis confirmed stable instrument performance via QC sample overlap. A total of 151 flavonoid-related metabolites were identified, but varied with culture durations. Metabolite counts were the highest at 80 days (143), followed by 60 days (142), and the lowest at 40 days (137). 129 metabolites were common across all samples, while others were culture-day specific (e.g., syringetin-3-O-glucoside at 40 days, apigenin 7-O-(6-O-malonylglucoside) at 80 days) (Fig. S2a).

**Fig. 3:**
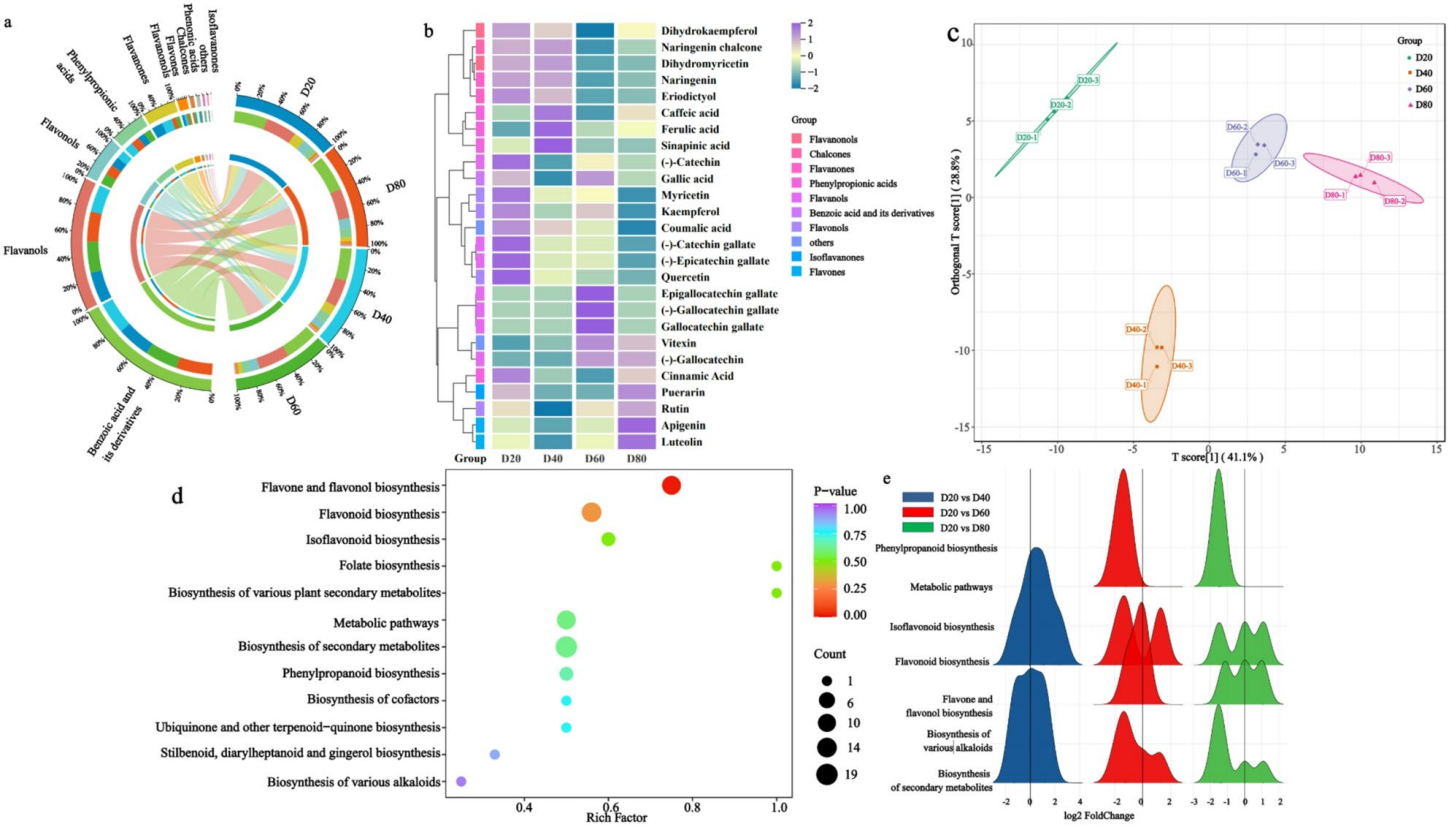
Analysis of flavonoid-related metabolism at different culture durations during adventitious shoot culture of *Cyclocarya paliurus*. **a** denotes the percentage and content analysis of every metabolite. The outer circle at the left denotes the category of all metabolites, the inner at the left denotes the percentage of metabolite at D20, D40, D60 and D80, respectively. The outer circle at the right denotes the content of all metabolites at D20, D40, D60 and D80, respectively; the inner at the right denotes the content of every metabolite at the same culture duration. **b** denotes heatmap analysis of various metabolites at different culture durations. **c** denotes Principal Component Analysis of different accumulated flavonoid-related metabolites (DAMs) at different culture durations. **d** denotes KEGG analysis of DAMs in all samples. The bigger the circle, the more DAMs on this pathway. **e** denotes KEGG pathway of DAMs at D40, D60 and D80 in comparison to D20. D20, D40, D60 and D80 denote adventitious shoot culture at 20 days, 40 days, 60 days and 80 days, respectively

Metabolites were classified into 11 categories: flavonols (28.48%), flavones (18.54%), flavanones (10.60%), benzoic acid and its derivatives (6.62%), flavanols (5.96%), flavanonols (5.30%), isoflavanones (4.64%), and others (Fig. 3a, Fig. S2b). And all of them further clustered into three subgroups with distinct temporal trends (Fig. S2c): Group 1 containing phenolic acids, chalcones, flavanonols and flavanones showed a gradual-decrease trend; Group 2 including flavanols, flavonols and others showed an initial decrease and then an increase; Group 3 containing flavones, isoflavanones, benzoic acid and its derivatives and phenylpropionic acids had a gradual increasing change with prolonged culture duration.

Among 11 categories of metabolites, benzoic acid derivatives had the highest content, followed by flavanols; isoflavanones content was the lowest (Fig. 3a). However, individual metabolite content varied with culture durations (Fig. 3b). Catechin (flavanols) had the highest content (> 1000 µg/g), showing a decrease followed by an increase with prolonged culture duration. Eriodictyol (flavanones) showed the lowest content at 60 days, being only 0.26 times (20 days), 0.31 times (40 days) and 0.71 times (80 days) compared to other durations. Afzelin (flavonols) decreased by 10.48% (40 days), then increased by 62.16% (60 days) and 44.84% (80 days) compared to 20 days. Vanillic acid (benzoic acid ant its derivatives) remained stable, while 4-hydroxybenzoic acid (benzoic acid and its derivatives) increased significantly from 20 to 80 days.

### Differentially accumulated metabolites (DAMs) variations at different culture durations

DAMs accounted for 47.37% of all metabolites. Principal Component Analysis revealed that flavonoid profiles at 60 and 80 days were similar, but distinct from those at 20 and 40 days (Fig. 3c). Compared to 20 days, 24 (9 up, 15 down), 33 (17 up, 16 down), and 39 (25 up, 14 down) DAMs were identified at 40, 60, and 80 days, respectively.

DAMs were enriched in three KEGG pathways, including biosynthesis of secondary metabolites (73.08%), flavone and flavonol biosynthesis (53.85%), and flavonoid biosynthesis (46.15%) (Fig. S2d). But, flavone and flavonol biosynthesis showed stronger enrichment than flavonoid biosynthesis and biosynthesis of secondary metabolites (Fig. 3d). The enrichment levels varied with culture durations: at 40 days: 100% DAMs were annotated to flavonoid biosynthesis, at 60 days: it was 55.56%, and at 80 days: it decreased to 33.33% compared to 20 days (Fig. 3e).

### Relationships among parameters

**Fig. 4:**
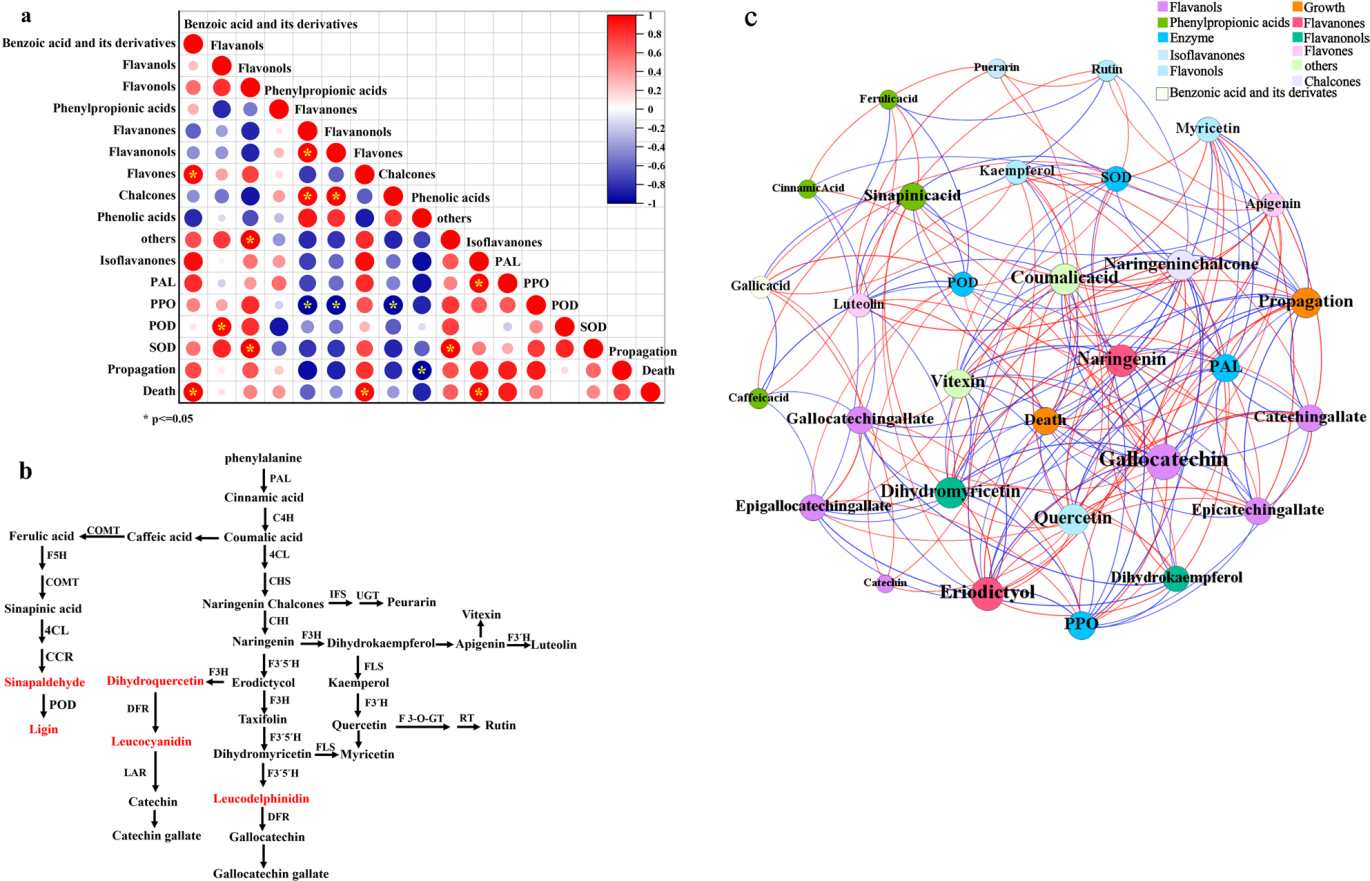
Relationship analysis between shoot propagation, enzymes and flavonoid-related metabolites. **a** means Pearson’s relationship among various metabolic groups, enzymatic activity and growth index. * denotes significant relationship between each other at the level of 0.05. Red line denotes positive relationship between each other, blue line denotes negative relationship between each other. **b** means a modal of flavonoid synthesis passway in shoot culture. Compounds with red color mean no detection in all samples. **c** means co-occurrence network analysis between flavonoid-related metabolites, enzymatic activity and growth index. Red line denotes positive correlation between each other, blue line denotes negative correlation between each other. Different circle size indicates the degree of correlation, the bigger the size is, the stronger degree exists

Significant relationships were observed among metabolite groups, enzymatic activities, and shoot growth. Adventitious shoot propagation showed a negative correlation with phenolic acids, but positive correlations with PAL, PPO, and POD. Adventitious shoot death exhibited a positive correlation with flavones (Fig. 4a). Metabolite expression within the flavonoid biosynthesis pathway revealed intrinsic interconnections (Fig. 4b). Co-occurrence network analysis showed that 99.47% of relationships were significant, with 50.26% being positive (Fig. 4c). For example, PAL had a positive relation with naringenin, a high level of POD took part in the transformation of sinapaldehyde to lignin. Quercetin had a significantly positive relation with catechin, but a negative relation with PPO. Vitexin had a positive relation with adventitious shoot propagation, but negative relation with adventitious shoot death.

## Discussion

Explant station has been proven to affect the control of explant contamination [7, 28]. Juvenile stems are usually used as explants due to their lower contamination rate, and quicker in vitro response compared to mature stems [7]. Contradictory with this result, juvenile stems of *C. paliurus* without lignification tend to brown easily and only contain one shoot compared to mature stems containing 4–5 of shoots in this study (Fig. 1a). Hence, mature stems are more suitable for use as explants in tissue culture compared to juvenile stems. In addition, explant source plays an important role on the success of explant sterilization [7]. Previous studies have demonstrated that shoot nodes from field-grown plants exhibit higher contamination rates [16, 28], further illustrated by the result of 100% shoot contamination in this study (Fig. 2a).

Various pretreatment methods have been applied to enhance disinfection efficiency in plant tissue culture. For example, Mukherjee et al. [12]. reported that an effective sterilization procedure for nodal segments of *Ramie* was that segments with 8–10 cm in length were kept in an ice-cold antioxidant solution, then washed under running water, and finally experienced multistage sterilization. But, some previous studies [7, 13] have proven that heat treatment is an effective method for producing microbe-free plants during the initial culture. In this study, a simple pretreatment involving cold storage at 4 °C for 8 days followed by water immersion at 37 °C also significantly reduced explant contamination to 12.27% (Fig. 2). It is possible that low-temperature storage formed stress and further induced various physiological and biochemical reactions in plants [29], while immersion at 37 °C-water disrupted microbial replication and dissemination, resulting in their inactivation [30, 31].

Sterilization duration is crucial for balancing disinfection efficacy and explant viability. Excessive exposure to sterilizing agents can damage tissues, while insufficient exposure may fail to eliminate contaminants [15]. This study confirmed that a 5-min treatment with HgCl₂ was optimal (Fig. 2c). Shorter durations were ineffective against deeply embedded pathogens, consistent with findings by Punja et al. [10]. However, longer durations increased stem contamination rates from 7 to 11 min without tissue damage in this study. This could be explained that many bioactive compounds in stem could hinder HgCl₂ from entering stems, or form complexes with HgCl₂ to weaken its sterilization effect. Notably, the sterilization solution became yellower from 7 to 11 min, suggesting the release of bioactive compounds from mature stems. A similar result was reported by Bassil et al. [32] showing that excessive sterilization can lead to the accumulation of phenolic compounds.

Plant growth regulators (PGRs) are crucial at every stage of tissue culture [7, 16]. In this study, GA₃ enhanced shoot elongation and development (Table 3, Fig. S1), consistent with previous finding that GA in combination with cytokinins stimulated growth [17]. Thidiazuron (TDZ), a synthetic cytokinin-like compound, is widely used to induce adventitious shoots in various species [16], further supported by the finding in this study (Table 2). Qu et al. [33]. demonstrated that the combination of TDZ and NAA promoted shoot proliferation in *Taxus chinensis*. Its effectiveness may be attributed to its auxin-like properties and impact on endogenous auxin metabolism [34].

However, plant species respond differently to PGRs concentration. Numerous studies have demonstrated that optimal ranges of PGRs promote shoot development and propagation, while concentrations outside these ranges inhibit growth [15]. For instance, Cheng et al. [8] found that high concentrations of 6-BA (4.0–12.0 mg/L) induced shoot formation at rates of 82.33%–96.67%, whereas this study found 51.04% induction using lower concentrations. This discrepancy may be due to differences in explant maturity. Cheng et al. [8] used nodal stems at the growth stage, while this study used fully lignified mature stems, which are less responsive to shoot induction [16]. In addition, adding 0.5 mg/L 6-BA, 1.5 mg/L IBA, and 0.03 mg/L TIBA to MS medium yielded a shoot multiplication coefficient of 3.20 for *C. paliurus* [8]. However, in this study, incorporating of 0.5 mg/L 6-BA, 0.01 mg/L TDZ, 0.3 mg/L GA₃, and 0.02 mg/L IBA into ½ MS achieved a propagation coefficient of 5.91, demonstrating superior performance compared to the media recommended by Cheng et al. [8] and Wu et al. [6] for shoot propagation.

Culture duration plays a critical role in promoting adventitious shoot growth. In this study, a 60-day culture duration was optimal for adventitious shoot propagation (Table 5). Exceeding 60 days of culture duration was unfit to adventitious shoot growth, resulting in adventitious shoot death (Fig. 1e). It is speculated that prolonged exposure to PGRs could stimulate the production of endogenous hormones or phenolic compounds, which could lead to shoot death or abscission [19, 20]. This was further inferred from higher SOD activity detected with extended culture durations (Table 5; Fig. 4c).

Enhanced shoot propagation was closely associated with the regulation of enzymatic activity (Fig. 4a and c). Superoxide dismutase (SOD), a key antioxidant enzyme, acts as the first line of defense against reactive oxygen species (ROS). During subculture, mechanical injury from shoot cutting induced ROS accumulation, leading to oxidative damage to cell membranes [35]. SOD activity was observed to increase with prolonged culture duration, suggesting its protective role in maintaining membrane integrity, as further evidenced by the result of co-occurrence network analysis (Fig. 4c). Phenylalanine ammonialyase (PAL) is a pivotal enzyme in the anthocyanin biosynthesis pathway, responsible for catalyzing phenylalanine to cinnamic acid [36]. This relationship was further highlighted by a positive associatioin between PAL and naringenin chalcones (Fig. 4c). Polyphenol oxidase (PPO) facilitates the oxidation of proanthocyanidins (PAs) into quinones, especially in response to wound stress [37], finally leading to plant browning or death. In this study, a significant negative relationship existed between PPO and epicatechingallate. PPO exhibited a positive with adventitious shoot propagation, but a negative correlation with adventitious shoot death (Fig. 4c). Peroxidase (POD) contributes to lignin biosynthesis by oxidizing ferulic acid into quinones. The lignification observed at the stem base with extended culture indicated the elevated POD activity linked to the conversion of sinapaldehyde to lignin (Fig. 4b).

Flavonoid-related metabolomics revealed that multiple biosynthetic pathways were active throughout the adventitious shoot culture (Fig. 4b and c). Phenylpropionic acids such as cinnamic and coumaric acid were converted into naringenin chalcone via enzymes including PAL, C4H, 4CL, and CHS. Subsequent transformations led to diverse flavonoid compounds. For instance, naringenin chalcone was converted to puerarin via isoflavone synthase (IFS) and UDP-glucuronosyltransferase (UGT), while dihydrokaempferol was further metabolized into luteolin, rutin, or myricetin through enzymes such as FLS and F3′H. Naringenin also served as a precursor for catechin derivatives, which were oxidized into quinonoid compounds by PPO.

Flavonoids, known for their antioxidant properties, are stored as bioactive compounds or polymerized onto cell walls [38, 39]. Their accumulation contributed to mitigate damage from mechanical injury and support adventitious shoot propagation, further demonstrating the result of co-occurrence network analysis in this study (Fig. 4c). Additionally, flavonols were the most abundant in terms of metabolite number, while flavanols exhibited the highest content. This differs from previous findings [38], possibly because of species-specific variations in flavonoid profiles. Various relationships were observed between each flavonoid compound and shoot propagation (Fig. 4c), consistent with previous studies [38, 40].

Notably, catechin (a flavanol) was enriched across all samples, predominantly in non-galloylated forms such as catechin and gallocatechin (Fig. 3b). This aligned with earlier reports of high catechin levels in *C. paliurus* leaves [41]. The galloylation of catechin resulted in the formation of catechin gallate and gallocatechin gallate, while epicatechin and epigallocatechin were absent, although their gallated forms were detected in samples with higher flavonoid content. This compound could be speculated to transform a new quinone under the regulation of PPO, further causing shoot death (Fig. 4c).

Root induction remains a challenge in *C. paliurus* tissue culture. Compared to previous studies [6, 8], root formation was limited, possibly due to genotype-specific responses. Additionally, plantlets failed to thrive in cultivation substrates, aligning with earlier reports [8]. This could be explained that incomplete root development could lead to a lack of nutrients and water supply to the conductive tissues, resulting in transplant failure of *C. paliurus* [8]. Additionally, a disruption in plant hormonal balances, such as ethylene and auxin [20], promotes leaf abscission observed during transplantation, further resulting in transplant failure. Auxin synthesis and transformation was blocked in leaf, further resulting in a decrease in auxin content in roots, finally inhibiting root growth [19]. Moreover, the change of growth environment could induce a stress, promoting the accumulation of H_2_O_2_ in plants [42]. Future research should focus on improving plantlet quality and acclimatization protocols to enhance survival rates.

## Conclusion

To develop an efficient system for mature stem disinfection and adventitious shoot propagation in *Cyclocarya paliurus*, this study explored various sterilization protocols and PGRs treatments. By treating mature stems with “lower-temperature storage and warm-water immersion”, the initial culture was efficiently improved, and an innovative solution was offered to alleviating a persistent problem during tissue culture. The suitable combination of 6-BA, TDZ, GA₃, and IBA complemented a high propagation coefficient (5.91-fold) with a critical time point of 60 days in shoot propagation culture. Correlations between shoot growth, enzymatic activities and flavonoid metabolites suggested a high level of SOD collaborated with various flavonoid metabolites to regulate shoot growth, furthermore, flavonoid metabolites exhibited variable roles in shoot propagation and death under the regulation of PAL, PPO, and POD through multiple flavonoid biosynthetic pathways.

## Supplementary Information

Supplementary Material 1.

## Data Availability

The data that support the findings of this study are available on request from the corresponding author upon reasonable request.

## Abbreviations

MS: Murashige and Skoog medium
6-BA: 6-benzylaminopurine
TDZ: thidiazuron
IBA: indole-3-butyric acid
2,4-D: 2,4-dichlorophenoxyacetic acid
NAA: naphthaleneacetic acid
UPLC–MS: Ultra high performance liquid chromatography–electrospray ionization tandem mass spectrometry
SOD: Superoxide Dismutase
PPO: Polyphenol oxidase
POD: Peroxidase
PAL: Phenylalanine ammonia-lyase
C4H: Cinnamic acid 4-hydroxylase
4CL: 4 Coumarate CoA ligase
CHS: Chalcone synthase
CHI: Chalcone isomerase
F3H: Flavanone 3-hydroxylase
F3ʹ5ʹH: Flavonoid 3ʹ5ʹ-hydroxylase
F3ʹH: Flavonoid 3ʹ-monooxygenase
FLS: Flavonol synthase
UFGT: UDP-favonoid glucosyl transferase
DFR: Dihydrofavonol 4-reductase
LAR: Leucoanthocyanidin reductase
IFS: Isoflavone synthase
UGT: UDP-glucuronosyltransferase
