# Supplementary material for: Optimizing disinfection and adventitious shoot propagation in Cyclocarya paliurus stem tissue culture: enzymatic and flavonoid metabolomics insights
Source: BMC Plant Biol. 2026 Apr 1;26:822. doi: 10.1186/s12870-026-08646-z (PMC13154591; doi:10.1186/s12870-026-08646-z)
Supplement: Supplementary file 1 — Supplementary Material 1. [file 12870_2026_8646_MOESM1_ESM.docx]

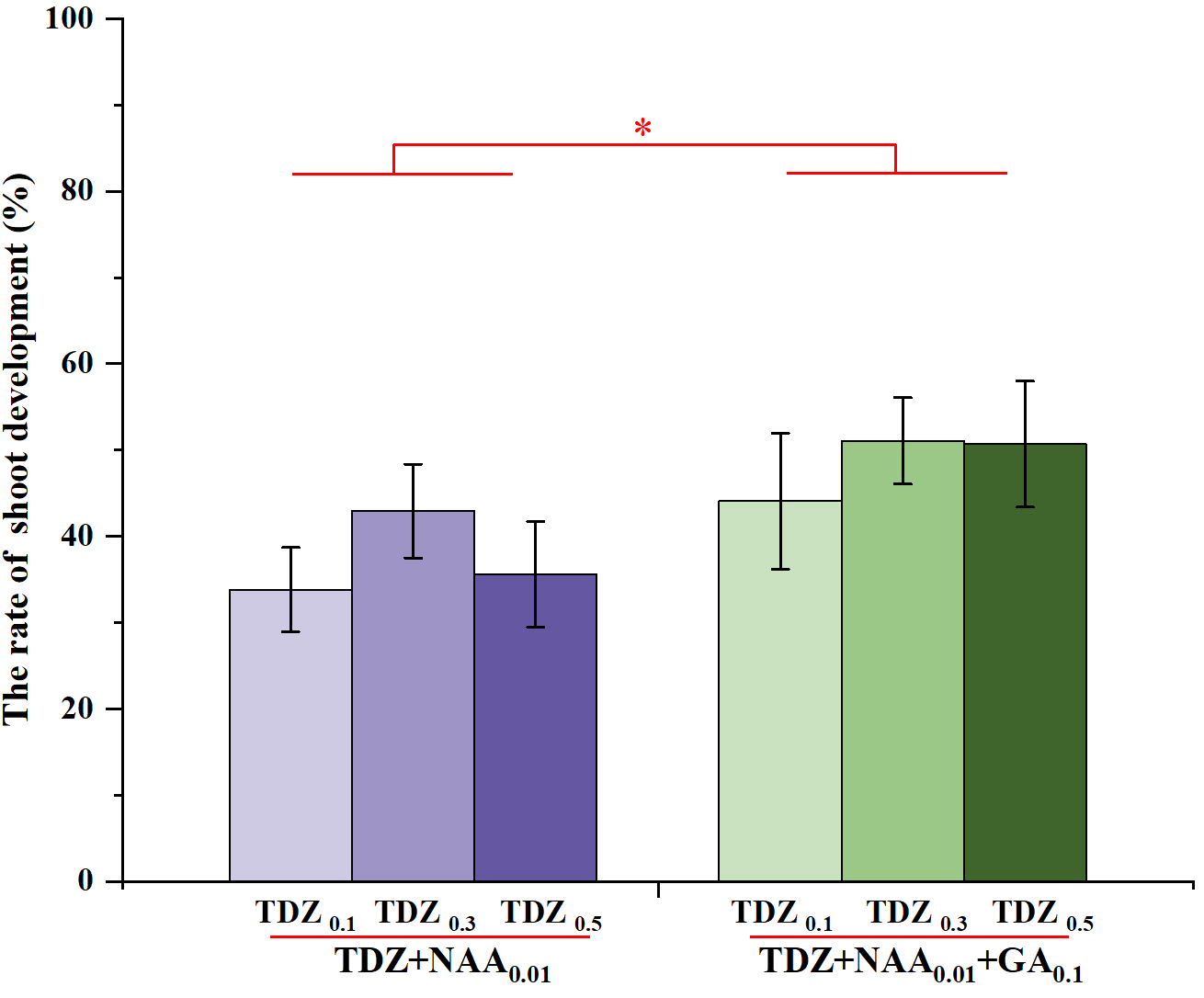


**Supplementary Fig. 1** The effect of different plant growth regulators on shoot development

Note: * denotes significant difference (α=0.05) between each other with Tukey’s HSD test for means.


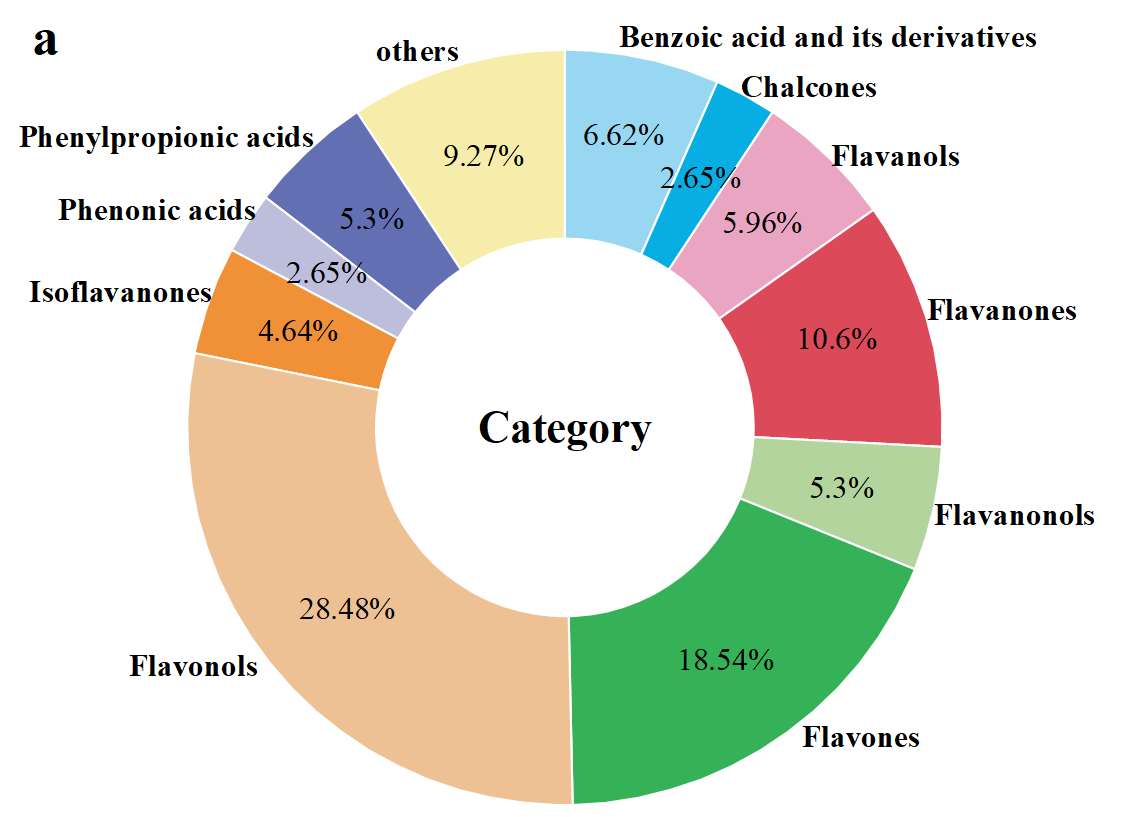

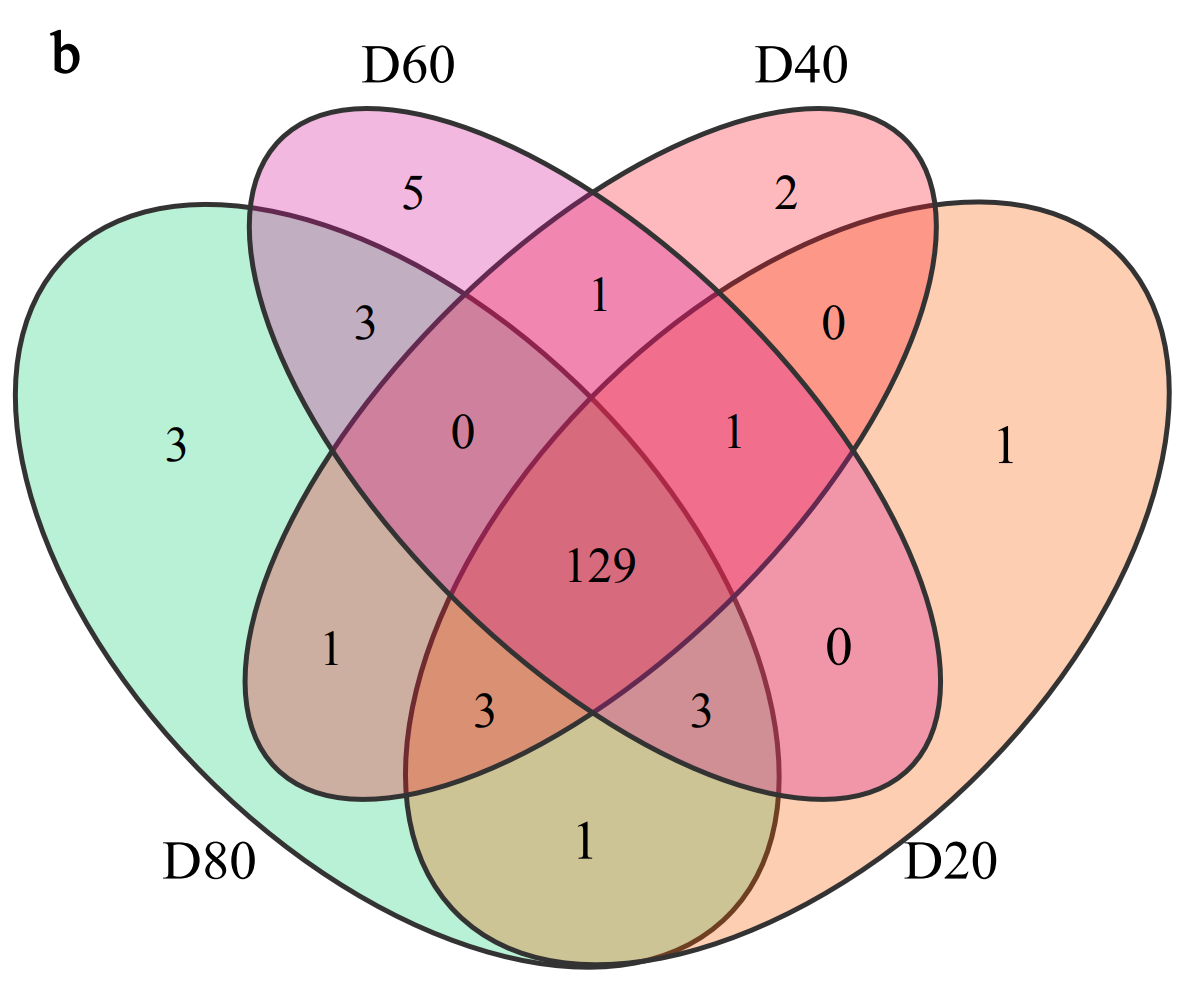


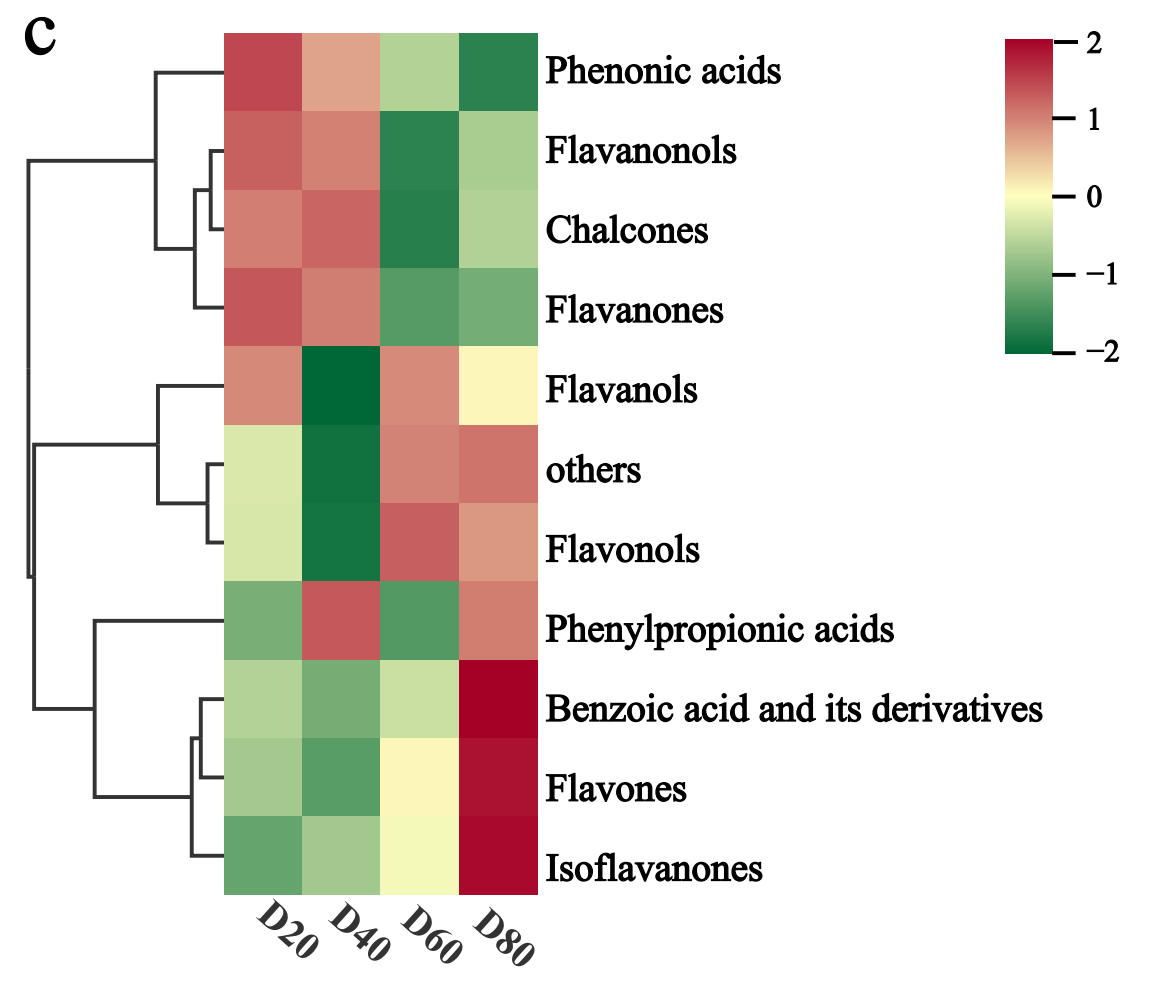

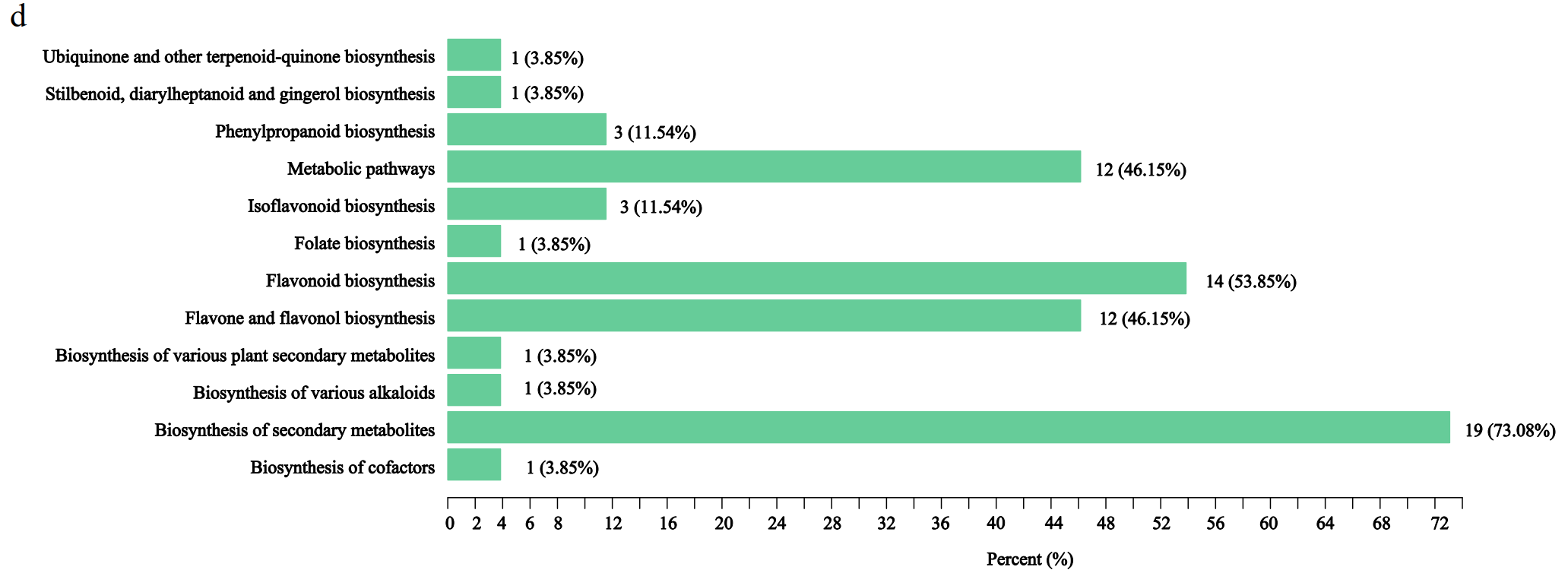


**Supplementary Fig. 2** Analysis of flavonoids-related metabolites of adventitious shoot at different culture durations

Note: a denotes the category of flavonoid-related metabolites detected in all samples. b denotes the number of metabolites detected in all samples. c denotes heatmaps of flavonoid-related metabolites. d denotes KEGG pathway analysis of metabolites. D20, D40, D60 and D80 denote adventitious shoot culture at 20 days, 40 days, 60 days and 80 days, respectively.
